# Indents

**Published:** 1921-01

**Authors:** 


### INDENTS

**☞Brief Articles of Technical or Scientific Value will receive notice and comment. Contributions invited.**

---

Lord Fisher's Maxims, Fear less—Hope more; Eat less—Chew more; Whine less—Breath more; Talk less—Say more; Hate less—Love more.

---

Pulp Removal. Pulp and peridental tissues once impaired by traumatism are never so resistent to infection as when in its normal condition. It is my opinion that injured pulps are capable of more serious consequences than small portions of the pulp left in root canals.—A. E. W., in *Oral Health.*

---

Bicarbonate of Soda for Sensitive Teeth. Rub a paste of soda with the fingers or a cotton pad over sensitive gums after scaling or when there is recession of the gums. A teaspoonful of soda in a half glass of warm water held in the mouth and rinsed about the mouth is very soothing after injuries from scaling or other painful operations.—L. B., in *Oral Health.*

Sensitive Dentine. Use a combination of chloroform, ether, and menthol by means of a hot air syringe. It will make tolerable many severe operations and will greatly relieve all painful operations.

---

Filling Small Cavities. Are cavities in carious teeth ever too small to be filled? As a matter of fact no cavity is too small that it may be left to grow large enough to make it difficult to fill. The filling that has the best fit, is the most perfectly shaped, and that presents the fewer stress angles, is the one that is likely to endure longest. The ideal is to prevent all cavities by prophylaxis and preventive caries. Next to prevention is early filling.

The First and Second District Dental Societies of New York recently unanimously passed this resolution: "Resolved, that the filling of initial cavities is a moral duty to our patients and is an obligation of our profession." So say we all of us. The best fillings are little ones, as the larger they are the greater the chance for failure. Why not hunt for the little ones and get them early? It is all wrong to allow small cavities to grow; in fact it is wrong to allow any cavity to get larger. Now is the time to fill, and treat, and prevent, and even to eradicate all the hopelessly diseased teeth, as we now know that dangerous infection may jeopardize the vital organs through the presence of bad teeth. There is DANGER in delay.—Extract from Editorial in *Oral Hygiene.*

---

Rustless Steel. In addressing the Central Association of German Dentists, Mr. Hauptmeyer, the director of Messrs. Krupp's dental clinic at Essen, paid tribute to the firm's institute for chemico-physical research, and particularly to the work of its president, Professor Strauss, the discoverer, in 1912, of an alloy of nickel, chrome and steel, offering a high degree of resistance to the attack of all kinds of acids. In the Baltic Exhibition at Malmö, in 1914, Krupp's rustless steel was put on the open market for the first time; but only since the war has it been available in bulk for general purposes. It is Mr. Hauptmeyer's opinion that this steel, or some modification of it, will entirely replace platinum and gold in dental mechanics. "Krupp's rustless steel offers the dentist a metal able to stand, both technically and hygienically, side by side with gold and platinum. It will take a high polish and it exhibits a high degree of resistance to all kinds of corrosion. In the matter of stability, it is superior to both of these metals; and it is considerably cheaper. Upon the application of this steel to dental mechanics will follow notable social developments in the world of dentistry. For the material is difficult to work and our laboratories must therefore largely substitute machinery for hand labor. Rustless steel can not be used to the fullest advantage without the help of the engineer. With his help, it should be possible, in the near future, to carry the working of steel into the smallest laboratory."—*Wiener Vierteljahrsschrift für Zahnheilkunde.*

---

ABNORMAL CALCIFICATION. The communication by Mr. F. W. Broderick in the *British Dental Journal,* October 15th, on "The Effect of Endocrine Derangement on the Teeth," is sure to attract the attention of all who are interested in the problem of caries causation. The ductless glands have hitherto been more than once brought up on suspicion and sometimes matters have gone so far as the framing against them of a formal charge of complicity in the destruction of teeth by caries. But Mr. Broderick has gone further than that and with surer stepping, and his bold and painstaking researches may be said to have prepared the way for the gathering and marshalling of such a body of evidence as may possibly and eventually constitute a clear and provable case against the ductless glands. It is pretty generally admitted that the teeth of some individuals, at different periods and while apparently sound, vary in the physical quality of hardness, and that in some cases the changes may be accompanied with variations in the composition of the saliva. But in the case of sound, fully-formed crowns, no one has yet been able to prove that these variations in hardness are caused by (or even associated with) a reduction in, or an addition to, the total calcium content of the tooth crown—except indeed where there is a clearly-recognized onset of one or other of the diseases known as caries and erosion. The coming and going of minute quantities of calcium, in response to some supposed conflict between the demand and supply of that element in the bodily economy, is too easy an assumption, and in the calcareous structure of the tooth it may well be that there are other substances, conditions, or vital processes that in maintaining hardness and resistance play a part that is quite as decisive as mere total weight of calcium present at each particular moment.—*Dental Record.*

---

Caution for Radical Operations. When you have made your examination, do not say, "We are ready to clean out the mouth." Have a physician examine that case from head to heel. He may find things you never dreamed of. Sometimes he will say, "Do not do anything. The patient can not stand the shock. Wait a few days, get him in proper condition." He checks him up again, and will tell you to begin very slowly.

Sometimes we are called into consultation by the physician, and we have not studied the case much, and we really aren't of very much help. They have to assume the responsibility, and so they say, "We want certain teeth taken out." The physicians will not take us into consultation if we can not render them assistance.

Do not promise your patients too much. You do not know the tissue change that has taken place in the joint or muscle, or in the heart valve or muscle, from streptococcal infection. You don't know what part your mouth infection has played. If we tell the patient his rheumatic condition will get all right, it may or it may not. We should tell him we hope to do some good and believe we can offer some help. But with an incurable condition in the mouth, teeth that can not be treated and brought back to a condition of health, you should be sure not to promise too much. The chronicity established oftentimes in tissues comes to such a point that Nature can not restore them to normal, and you can not bring those tissues back to a condition of health.—Dr. R. Boyd Bogle, in *Dental Summary.*

---

EULOGY OF HENRY W. MORGAN, D.D.S. The Tennessee State Dental Association has had from the time of its organiaztion some of the very best men to be found in the country. Here we pause for a moment and think of our much beloved and grand old man, Dr. Henry W. Morgan, a man who missed so few state meetings in all his useful career, and at our last meeting, while he was deprived of being present on account of sickness, sent us a telegram of "greetings." While he has crossed the River, yet he leaves an influence that will live for generations and a memory in our hearts which will be forever green. From the lives of older men who have gone before us, we learn many great and true lessons. That which makes a man great and beloved in any community or any group, is the devotion and sincerity with which he enters into his work. We are all endowed by the Great Maker with certain talents, and it is our duty to use these talents and multipy them and not bury them like the foolish servant. In the life of Dr. Morgan we see an example of the man who used his talents and multiplied them, an example of a man who devoted his time, his life, his all to the uplift and the advancement of the dental profession. And I am sure that every man within the sound of my voice gets from the example of Dr. Morgan an inspiration of ambition and of service.—Read before the Tennessee State Dental Society, May, 1920.

---

Development of Health Specialists. As a health man, I was especially interested in the proposal that in the school hygienist there should be combined the functions of the present dental hygienist and the function of school inspector, and perhaps in some measure the school nurse may combine some of the functions of the school teacher also.

The development of hygienists and the doing of this particular work will make more work for dentists rather than less, just as it makes more work for physicians to have preventive work done than it does not to have it done. The reason is not far to seek. The doing of this work establishes high standards, and high standards mean more work for those who are doing the kind of work necessary to meet these high standards. The best illustration we can have, and the one I usually make use of is the development of the eye specialist. A generation ago there were no eye specialists. Eyes were neglected. Man accepted the inevitable, weak vision, when he reached a certain time of life. Many men accepted it in a fatalistic spirit; it could not be avoided, and therefore it had to be endured. But there came the eye specialist who rendered distinct service. Man saw it was no longer necessary that he should accept a low standard, but he demanded a high standard of eye capacity, and there grew up a large, well-paid division of the medical profession—the eye specialists. Some day we will have the same standard for nose conditions, for skin conditions, for chest conditions, for scores of things I might mention. We have not enough medical men to meet that demand. You have discovered as dentists that as you have raised the standards of care of the teeth, you have not dentists enough to supply the demand for the service. The natural outcome of it will be that in your organizations you must work until you have highly trained men whose whole time will be occupied with this work. Around them will be an outer circle of people rendering a service that does not require the same degree of skill; around them there can be a cordon of trained nurses, a cordon of social workers, if you please. I am merely using these as illustrations. In other words, the time must come when, through organization, there will be a group of men who have expended their time and money for the acquirement of a high degree of skill, who will confine all their time to giving that amount of skill and be well paid for doing that which they can do better than anybody else can do it, and they will abandon doing things that can be done by anybody. When that time comes nobody will feel competition. They need not fear their newspaper doctor who works for two cents a day, or the other fellow who prescribes over the counter, the druggist, or any service of that type.

To my mind there is no question whatsoever but that the development of dental hygiene, with a trained corps of dental hygienists, is not only a necessity of the social situation, but from your standpoint, speaking of it now from a trade union point of view, it is well for you rather than otherwise. A particularly significant thing to my mind is this: After some four or five or six years of trial in the field of dental hygiene, from those men who have been in close touch with it we find them coming back to the idea that it is impossible to divorce the work of the medical and dental professions. We have got to train joint workers to work in dentistry and work in medicine. That is very interesting.

We have had a great deal of controversy in the field of preventive medicine as to just what should be the training that person should have. Years ago a few of our schools started in by beginning that training with the degree of Doctor of Philosophy in Medicine or in Public Health, requiring as the first essential that a man shall have had a medical degree and build two years thereon. There is need in this country for men trained in that way, but it is a small need. A very great need is for people trained in shorter courses, in what might be termed the art rather than the science of the profession of preventive medicine.—Dr. W. A. Evans, in *Journal N. D. A.* for June.

---

PAINLESS AND SANE DENTISTRY. A recent article in which Dr. C. N. Johnson treats of the baneful influence of fear in relation to our daily work, is most timely and thoroughly appreciated from our viewpoint. Habec has frequently made the statement that the dentist finds his most skillful services called for in the treatment of the patient above the eyebrows rather than below; in other words, dissipation of fear is essential before good service may be rendered.

We well recall a statement by Dr. Johnson relative to painless dentistry in which his lifelong experience has proven that pain can best be limited by the aid of small, sharp instruments, especially the bur, skillfully manipulated and which must also be accompanied by confidence in the operator. When this situation obtains, to which is added a spirit of co-operation and helpfulness by the patient, the best dentistry is accomplished. Never in the history of the profession was the safe and sane dentist more needed than at this time, while the spirit of devastation is rampant throughout the land. Nothing could be more apparent or indisputable than the fact that, through lack of comprehension of dental conditions from the standpoint of the dentist, our medical confreres are rapidly forcing us into an unparalleled period of destructive dentistry, and we regret to say that many of our own profession do not seem adverse to the situation. Those of us who have passed through one after another of these periods of revolution, as it were, and watched the short and violent course of each successive innovation, can more fully understand what it all means and very closely predict the final outcome. It is scarcely exaggeration to say progressive dentistry as an actual science has received a severe, though temporary, check. In fact, our vaunted skill in the treatment of diseased teeth is threatened to be classed as a Lost Art. We do, however, appreciate that, like the great events in history, from chaos a new structure rises far more useful and potent than the original. This fact we may cling to as our Great White Hope. We can, however, begin to see the light breaking through the clouds and the prospect for general improvement in our methods is apparent, and we will be able to approach the present problem with a clear understanding of cause and effect. We are gradually developing a large number of young men who will creditably follow the lead of those who are now filling the high places in our great profession.—"Habec," in *Oral Health.*

---

INSTRUCTION IN DENTAL HYGIENE. An institution or a community unit whose children's mouths are once put in order can be kept in good shape with a comparatively small effort, provided there is some one constantly on guard to see that the work goes on and that caries is checked before extensive damage is done.

I have several times made the statement that the battle for the child's mouth is lost or won by the age of eight. Under proper schooling in oral hygiene between the ages of two and eight years, if the mouth is kept in order to this age, very little dental work is needed after that time. My work has amply confirmed this statement. I am now caring for a community settlement of fifteen hundred children without assistance except from lay helpers and without remuneration. I am able to do this because the social service workers give me the most perfect co-operation.

One of our activities is a day nursery where we average one hundred and fifty children from the cradle to eight years. For two years I selected and observed fifty of these children with average mouths between the ages of two and four and we did not have a single case of caries in the two years. We used a systematic toothbrush drill and regular and periodical inspection of the mouths.

Education of the parent is essential; but there is no way to secure this instruction excepting through the children themselves at school.

I am more than ever convinced of the truth of this. I now wish to go a bit farther and ask you for a prerequisite to the instruction of the child. It is for the proper instruction of the dentist himself and the doctor in his school.

The medical profession today is asking us for a character of work which we, as a profession, are not prepared to give, because of defective training in the care and management of the child himself. That the child's mouth is neglected is true; but we are partly to blame. The average dentist would gladly part with his child patient, if he were not to lose prestige thereby. As it is, he gives the child's mouth insufficient care because he dreads the encounter or perhaps lacks the patience or tact to put himself in proper relation with the child. He salves his conscience with the thought of the temperamental difficulties of the case and puts silver nitrate into carious first molars and lets them go on to destruction.

He has never had specific instruction in the art of managing the child and quite often only a superficial attempt at instruction in the treatment of the conditions encountered in the child's mouth which are peculiar to the age of childhood.

Can we expect to give to the medical profession the guidance which it asks for, if we neglect in our dental colleges the teaching of this branch?

My opinion is that the object we all wish for—namely, the proper care of the child's mouth—is to be obtained by:

1. Systematic and regular dental inspection in the schools;
2. Instruction of the child and his teachers in oral hygiene;
3. Employment of full-time dentists, who themselves have received a special training in the work;
4. Employment of a force of social service workers whose duty it shall be to seek the co-operation of the parent. This is to be done by visiting the homes, by organization of mothers' clubs and keeping up an interest in their meetings.

This looks like a formidable program. It doubtless is an expensive one.—H. W. Guthrie, in *Journal N. D. A.*

---

Casting a Band or Crown. If I want to make a Carmichael, or a gold band, or a gold crown, I make a copper band and get my impression with the Taggart wax, allowing the patient to bite, and get the occlusion, chill and take impression. Then build up the root so as to handle it and cut off the band from the crown and invest the whole. Put a sprue in the Taggart wax, invest and cast. If I use a post, having it in place I take my impression, and it is in the wax of the impression. The original wax of the impression becomes my pattern, and I invest that, burn it out, and cast. If making a Carmichael, I use the copper shell with the top and that gives me condensation; then I slightly warm the copper band, when we invest and it comes away; then slightly moisten it, and it comes away easily. It is a very simple method, but we get wonderful results.—Dr. Conzette, in *Items of Interest.*

Oral Hygiene. The matter of the education of the public in regard to the necessity of a clean mouth and teeth should be emphasized in this day as never before, for with all of the knowledge that we now possess concerning the disastrous consequences attendant upon diseased conditions of the oral cavity, the necessity for oral hygiene and prophylaxis is magnified beyond any of our previous conceptions. We would advise a more emphatic campaign in public instruction by the Oral Hygiene Committee and would advise the passage of such legislation by the association that would stimulate the interest of the profession and through them the general public in this important subject. The funds necessary for the prosecution of the work of the committee should not be withheld, and anything that we, as an association, can do, should be done to further the work of this committee. No matter with which we have to deal is of more importance. We can not hope to cope with the problem of oral health by the measures which we have been pursuing in the past. Tooth decay and pyorrhea are advancing far more rapidly than are the remedial measures which we are instituting, and the only hope for the future lies in the prevention of these diseases through the instrumentality of a knowledge of the beneficial effects of a clean mouth. The constitutional amendment upon that subject, which will come up for passage at this session, should receive the approbation of the delegates and be unanimously carried.—*Journal N. D. A.*

